# Association of Gout with Head and Neck Cancer: Longitudinal Follow-Up Studies Using a National Health Insurance Database in South Korea

**DOI:** 10.3390/jcm13113136

**Published:** 2024-05-27

**Authors:** So Young Kim, Il Hwan Park, Chun Sung Byun, Hyo Geun Choi, Mi Jung Kwon, Ji Hee Kim, Joo-Hee Kim, Chang Wan Kim

**Affiliations:** 1 Department of Anatomy and Cell Biology, Seoul National University College of Medicine, Seoul 03080, Republic of Korea; sossi81@snu.ac.kr; 2 Department of Cardiovascular and Thoracic Surgery, Yonsei University Wonju College of Medicine, Wonju 26426, Republic of Korea; nicecs@yonsei.ac.kr (I.H.P.); csbyun@yonsei.ac.kr (C.S.B.); 3 Suseoseoulent Clinic, Seoul 06349, Republic of Korea; pupen@naver.com; 4 Department of Pathology, Hallym University Sacred Heart Hospital, Hallym University College of Medicine, Anyang 14068, Republic of Korea; mulank99@hallym.or.kr; 5 Department of Neurosurgery, Hallym University Sacred Heart Hospital, Hallym University College of Medicine, Anyang 14068, Republic of Korea; kimjihee@hallym.or.kr; 6 Department of Medicine, Hallym University Sacred Heart Hospital, Hallym University College of Medicine, Anyang 14068, Republic of Korea; luxjhee@hallym.or.kr

**Keywords:** gout, head and neck cancer, risk factors, case–control studies, epidemiology

## Abstract

**Objective**: Previous studies have reported controversial results on the association between gout and the risk of cancer. This study aimed to investigate the relationship between gout and the incidence of head and neck cancer (HNC). **Methods**: The data of participants who underwent health checkups in 2009 were analyzed using the National Health Insurance Database in South Korea. A total of 14,348 HNC patients and 57,392 control participants were analyzed for a prior history of gout. Overlap weighting was applied, and odds ratios (ORs) of gout for HNC patients were analyzed. The overlap-weighted model adjusted for demographic, socioeconomic, and lifestyle factors and comorbidities. HNC sites were classified as oral cavity cancer, oropharyngeal cancer, nasopharyngeal cancer, hypopharyngeal cancer, nasal cavity/sinus cancer, larynx cancer, or salivary gland cancer, and the ORs of gout were estimated for each site. **Results**: Overall, patients with HNC had 1.12-fold greater odds of having gout (95% confidence intervals [CIs] = 1.04–1.20). According to the site of HNC, oral cavity cancer, oropharynx cancer, and larynx cancer demonstrated high odds of having gout (OR = 1.25, 95% CI = 1.16–1.34 for oral cavity cancer; OR = 1.08, 95% CI = 1.01–1.15 for oropharynx cancer; and OR = 1.12, 95% CI = 1.06–1.20 for larynx cancer). On the other hand, nasal cavity/sinus cancer, nasopharynx cancer, and salivary gland cancer presented low odds of having gout (OR = 0.78, 95% CI = 0.72–0.84 for nasal cavity/sinus cancer; OR = 0.89, 95% CI = 0.83–0.96 for nasopharynx cancer; and OR = 0.88, 95% CI = 0.81–0.96 for salivary gland cancer). **Conclusions**: A prior history of gout was associated with a high overall incidence of HNC. Oral cavity cancer, oropharynx cancer, and larynx cancer have a high incidence in gout patients. However, nasal cavity/sinus cancer, nasopharyngeal cancer, and salivary gland cancer have low incidences in gout patients. The impact of gout on HNC risk should be specifically considered according to the site of the HNC.

## 1. Introduction

Gout is a painful inflammatory arthritis caused by the formation of monosodium urate crystals in articular and nonarticular structures [1]. Approximately 1–4% of the worldwide population suffers from gout [2]. The prevalence of gout is greater in men than in women, with an estimated sex ratio of 3:1–10:1, and increases with age [2]. Long-term hyperuricemia can lead to the deposition of monosodium urate crystals, which in turn promote the activation of the NOD-like receptor protein 3 inflammasome and provoke gout flare [3]. Chronic inflammatory status and arthritis in gout patients may increase susceptibility to other diseases. Patients with gout have many comorbidities, such as hypertension, chronic kidney disease, and metabolic syndrome [2,4]. The bidirectional impact of uric acid, both antioxidant and pro-oxidant, can impose the risk of subsequent comorbidities in addition to the effect of inflammasome cascades initiated by monosodium urate crystals.

Several previous studies have reported the risk of cancer in patients with gout [5,6,7]. According to a nationwide health claim cohort study, patients with gout had a 1.053-fold greater risk of overall cancer (95% confidence intervals [CI] = 1.031–1.077) [5]. On the other hand, a few previous studies have reported no association between gout and the risk of cancer [8]. There was an additional risk of gastric cancer in the overall population and in secondary groups according to demographic factors and comorbidities [8]. Indeed, the risk of cancer related to the presence of gout is heterogeneous according to the type of cancer [5].

A prior study demonstrated that there was no association between gout and head and neck cancer (HNC). However, no prior study has analyzed the relationship between gout and HNC according to the site of cancer. In addition, because the risk of HNC is elevated in populations who smoke and consume alcohol, these lifestyle factors should be included in the analysis [9].

We hypothesized that the incidence of HNC could be greater in patients with gout. To test this hypothesis, we analyzed health claim data from patients with HNC in Korea with a previous history of gout. In addition, because there may be a specific association with gout according to the type of HNC, this study analyzed the HNC type and prior history of gout in each HNC subtype.

## 2. Methods

### 2.1. Ethics

The present study was permitted by the ethics committee of Hallym University (2021-02-004). The ethics committee exempted the requirement of written informed consent. This study was based on the National Health Insurance Database (NHID) in South Korea [10]. The NHID contains sociodemographic characteristics, health care utilization, dates of birth and death, and health screening.

### 2.2. Exposure and Outcomes

The history of gout was defined with the diagnosis of gout (ICD-10: M10) ≥ 2 times [11].

The history of head and neck cancer (HNC) (ICD-10: C00-C14, C300, C31–C32) with special certification for cancer (codes V193 and V194) was regarded as the history of HNC in this study.

In this study, we used ICD-10 codes to investigate the sites of the following eight types of head and neck cancer (HNC): oral cavity cancer (C00–C06), oropharynx cancer (C09–C10), nasopharynx cancer (C11), hypopharyngeal cancer (C12–C13), nasal cavity/sinus cancer (C300 and C31), laryngeal cancer (C32), and salivary gland cancer (C07–C08).

### 2.3. Participant Selection

**Figure 1:**
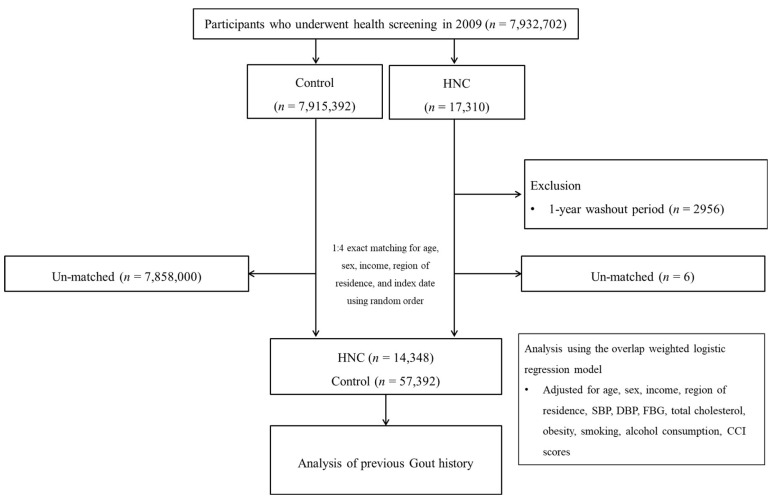
Schematic illustration of the participant selection process used in the present study. Of a total of 7,932,702 participants, 14,348 HNC participants and 57,392 control participants were included. (HNC: head and neck cancer; SBP: systolic blood pressure; DBP: diastolic blood pressure; FBG: fast blood glucose; CCI: Charlson Comorbidity Index).

For this study, we obtained a customized dataset of adults who underwent a health checkup in 2009 from the NHID in South Korea (*n* = 7,932,702). HNC participants were selected from the whole Korean population in the NHID from 2009 through 2021 (*n* = 17,310). The participants without history of HNC between 2009 and 2021 were control participants (*n* = 7,915,392). The HNC participants who were diagnosed in 2009 were removed (*n* = 2956). HNC participants and control participants were 1:4 matched for age, sex, income, and region of residence. Ultimately, 14,348 HNC participants and 57,392 control participants were enrolled in this study (Figure 1).

### 2.4. Covariables

This study included covariables of demographic, socio-economic, and lifestyle factors and comorbidities. Age was classified into 12 groups [12]. Tobacco smoking, alcohol consumption, and obesity according to BMI (body mass index, kg/m^2^) were categorized as described in our previous study [13]. Systolic blood pressure (SBP, mmHg), diastolic blood pressure (DBP, mmHg), fasting blood glucose (mg/dL), and total cholesterol (mg/dL) were measured parameters in the National Health Insurance system.

The Charlson Comorbidity Index (CCI) score was calculated [14,15]. This study excluded cancer and metastatic cancer patients from the CCI.

### 2.5. Statistics

The propensity score (PS) was estimated using multivariable logistic regression. HNC participants and control participants were applied the probability of PS and the probability of 1-PS, respectively. Overlap weighting was calculated [16,17].

The propensity score overlap-weighted logistic regression model was applied to estimate the overlap-weighted odds ratios (ORs) of gout for HNC patients. Additionally, the overlap-weighted ORs of gout for the site of HNC were analyzed. The age and sex subgroups were analyzed.

A *p*-value < 0.05 was considered as statistical significance. All analyses were conducted using SAS version 9.4 (SAS Institute Inc., Cary, NC, USA).

## 3. Results

Overall, 5.11% (733/14,348) of the HNC group and 4.66% (2674/57,392) of the control group had a previous history of gout (Table 1). The distributions of BMI group and smoking status; measurements of systemic blood pressure, diastolic blood pressure, fasting blood glucose, and total cholesterol; and CCI score differed between the HNC group and the control group. Thus, overlap-weighting adjustment was conducted. After overlap-weighting adjustment, 5.10% (577/11,300) of the HNC group and 4.60% (520/11,300) of the control group had a history of gout (sd = 0.02).

The odds of previous gout were 1.10-fold greater in the HNC group than in the control group according to the crude model (95% CI = 1.01–1.20, *p* = 0.024; Table 2). After overlap weighting, the odds of having gout for HNC patients were 1.12-fold greater in the HNC group (95% CI = 1.04–1.20, *p* = 0.002). Among the subtypes of HNC, oral cavity cancer had the greatest association with gout history (OR = 1.25, 95% CI = 1.16–1.34; *p* < 0.001). Oropharynx cancer and larynx cancer were also strongly associated with previous gout (OR = 1.08, 95% CI = 1.01–1.15, *p* = 0.029 for oropharynx cancer and OR = 1.12, 95% CI = 1.06–1.20, *p* < 0.001 for larynx cancer). On the other hand, nasal cavity/sinus cancer had the lowest odds of being related to a previous history of gout (OR = 0.78, 95% CI = 0.72–0.84; *p* < 0.001). Nasopharyngeal cancer and salivary gland cancer were also weakly associated with a previous history of gout (OR = 0.89, 95% CI = 0.83–0.96; *p* = 0.003 for nasopharyngeal cancer; OR = 0.88, 95% CI = 0.81–0.96; *p* = 0.002 for salivary gland cancer).

**Figure 2:**
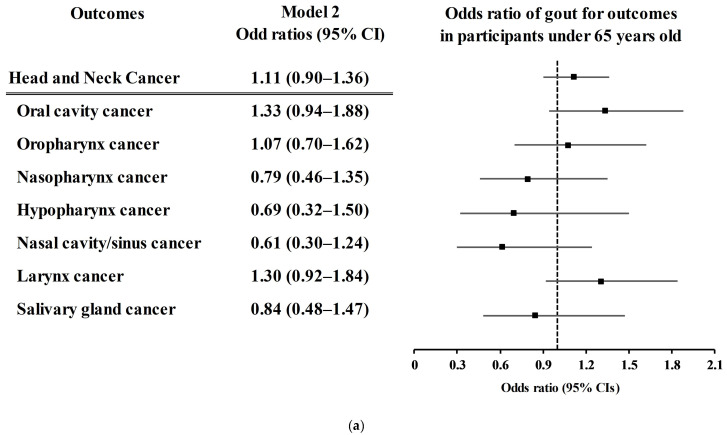

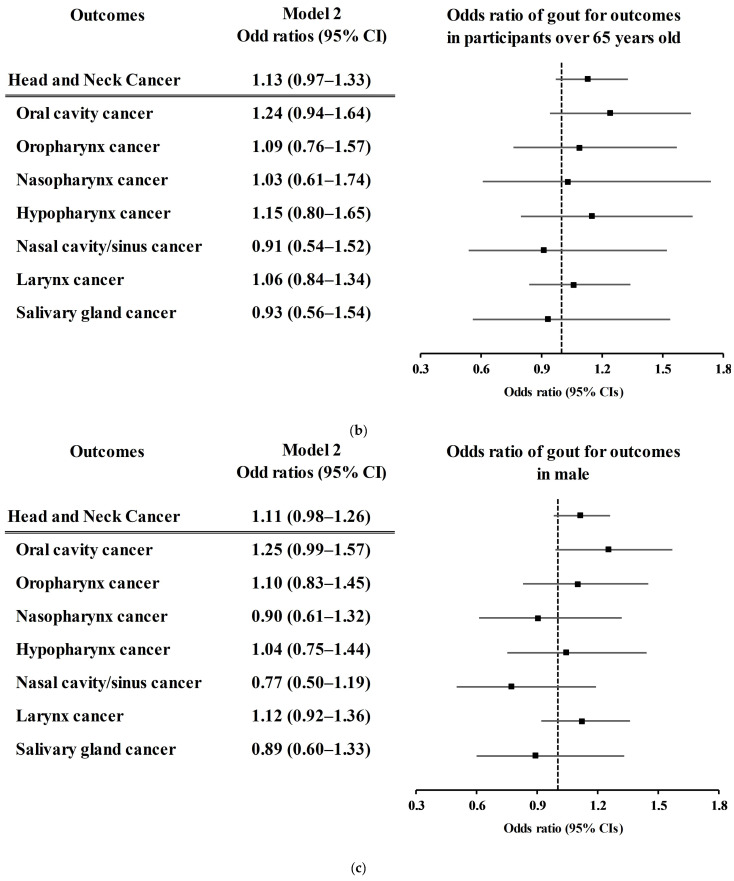

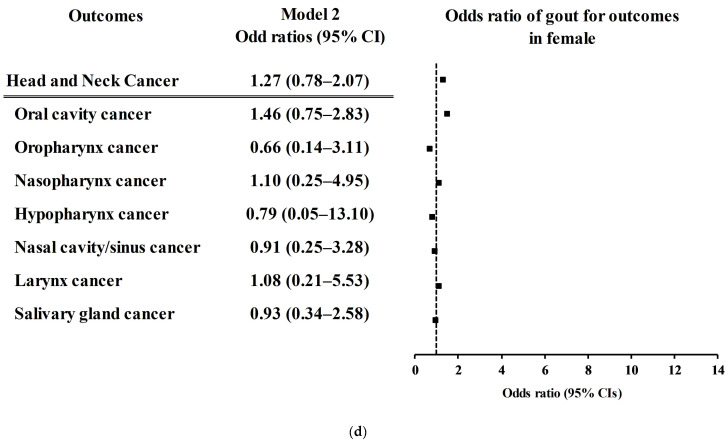
(**a**). Odds ratios of gout for head and neck cancer subtypes in participants under 65 years old. (**b**). Odds ratios of gout for head and neck cancer subtypes in participants over 65 years old. (**c**). Odds ratios of gout for head and neck cancer subtypes in males. (**d**). Odds ratios of gout for head and neck cancer subtypes in females.

The associations of each type of HNC with previous gout history according to age and sex subgroup were analyzed (Figure 2 and Tables S1–S7). There was no association of previous gout history with the incidence of overall HNC, oral cavity cancer, oropharynx cancer, nasopharynx cancer, hypopharynx cancer, nasal cavity/sinus cancer, larynx cancer, or salivary gland cancer in any of the age or sex subgroups (Figure 2 and Tables S1–S7).

## 4. Discussion

A previous history of gout was related to a higher incidence of HNC in the Korean population. Oral cavity cancer had the strongest association with previous gout history, followed by laryngeal cancer and oropharynx cancer. On the other hand, nasal cavity/sinus cancer had a lower incidence in gout patients, followed by nasopharynx cancer and salivary gland cancer. This study improved upon previous findings by revealing an association between a history of prior gout and HNC, especially for each type of HNC.

Previous studies have shown that gout patients have a greater incidence of various types of cancer [5,18,19]. In a longitudinal population cohort study, patients with gout demonstrated an increased risk of esophageal cancer, stomach cancer, colon cancer, liver cancer, pancreatic cancer, lung cancer, ovarian cancer, renal cancer, and bladder cancer [5]. Another follow-up cohort study reported high hazard ratios for prostate cancer, bladder cancer, and renal cancer in patients with gout [19]. The potential risk of HNC in gout patients has shown mixed results in previous studies [5,18,19]. In a prospective cohort study, the incidence ratio of oral and pharyngeal cancer was 1.48–1.92 in patients with gout, although the confidence intervals were wide [18]. However, other longitudinal cohort studies did not show a high risk of HNC in patients with gout [5]. Because smoking and alcohol consumption are potent risk factors for HNC, the effects of these factors on the association between gout and HNC should be considered [20]. In the present study, we considered lifestyle factors, including smoking, alcohol consumption and socioeconomic factors, which are known to increase the risk of HNC [21,22].

Hyperuricemia in gout patients can mediate the high incidence of HNC in gout patients [23]. A meta-analysis demonstrated that the pooled risk for cancer incidence was 1.08 (95% CI = 1.04–1.12), and the risk of cancer mortality was 1.15 (95% CI = 1.05–1.26) [23]. A prospective study revealed that high serum uric acid increased the risk of mortality from cancer (adjusted hazard ratio = 1.41, 95% CI = 1.22–1.62) [24]. Hyperuricemia can be attributed to the high nucleic acid turnover and proliferation of cancer cells [24]. Moreover, chronic systemic inflammation in gout patients can impose a burden on the subsequent incidence of cancer.

On the other hand, nasal cavity/sinus cancer, nasopharyngeal cancer, and salivary gland cancer did not have a high incidence in gout patients in this study. The antioxidant effects of uric acid may be linked to the low risk of nasal cavity/sinus cancer, nasopharyngeal cancer, and salivary gland cancer in our cohort [25]. Uric acid scavenges reactive oxygen species (ROS) during its conversion to allantoin [25]. Furthermore, because a considerable portion of gout patients are prescribed allopurinol, the protective effects of allopurinol on cancer can lower the incidence of cancer in gout patients. In addition to lowering urate, allopurinol reportedly inhibits ROS, tumor necrosis factor alpha, and NLRP3 activities [26]. Thus, studies on the effects of gout and gout-related medications on the incidence of cancers can yield mixed results. For nasopharyngeal cancer, seropositivity for Epstein‒Barr virus (EBV) and exposure to some carcinogenic chemicals have crucial impacts on the incidence of this disease [27]. For nasal cavity cancer, exposure through the nasal cavity mucosa to carcinoid chemicals can increase the risk of incidence [28]. Therefore, the impacts of gout on the nasopharynx or nasal cavity can be diluted due to other potent risks, such as seropositivity to EBV and chemical exposure [29].

The present study analyzed data from a large, nationwide cohort. The large size of the study population permitted unbiased selection of the control population. In addition, we applied an overlap-weighted model to attenuate the confounding effects from covariables. However, because this study was based on national health claim codes, patients with subclinical cases or missed diagnoses who did not visit clinics were excluded. It was also possible that diagnostic codes could be registered incorrectly due to insufficient evaluation in clinics. In addition, the pathologic types, staging, and treatment options of HNC could not be included in the analyses [30,31,32]. The types, severity, and management of gout were also not considered in the present study. Although this study used a huge study population, the cases of specific locations of HNC could be too small to show the statistically significant relations. Because this study was based on a Korean population, the current results need to be interpreted with consideration of ethnic and geographic circumstances. Finally, we investigated the previous history of gout before the diagnosis of HNC, and we could not determine the causal relationship between gout and HNC. Further studies are warranted to address these limitations.

## 5. Conclusions

Patients with gout have greater odds of having HNC. Oral cavity cancer, oropharynx cancer, and larynx cancer were associated with a previous history of gout. On the other hand, nasal cavity/sinus cancer, nasopharyngeal cancer, and salivary gland cancer had lower incidences in gout patients. In the clinic, patients with gout need to be managed considering the potential risk of HNC.

## Figures and Tables

**Table 1:** General Characteristics of participants after and before overlap propensity score weight.

Characteristics	Before Overlap-Weighting Adjustment			After Overlap-Weighting Adjustment		
	HNC	Control	Standardized Difference	HNC	Control	Standardized Difference
Age (n, %)			0.00			0.00
20–24	3 (0.02)	12 (0.02)		2 (0.02)	2 (0.02)	
25–29	26 (0.18)	104 (0.18)		21 (0.18)	21 (0.18)	
30–34	126 (0.88)	504 (0.88)		100 (0.89)	100 (0.89)	
35–39	270 (1.88)	1080 (1.88)		215 (1.90)	215 (1.90)	
40–44	418 (2.91)	1672 (2.91)		332 (2.93)	332 (2.93)	
45–49	716 (4.99)	2864 (4.99)		567 (5.01)	567 (5.01)	
50–54	1318 (9.19)	5272 (9.19)		1039 (9.19)	1039 (9.19)	
55–59	1840 (12.82)	7360 (12.82)		1445 (12.79)	1445 (12.79)	
60–64	2158 (15.04)	8632 (15.04)		1692 (14.98)	1692 (14.98)	
65–69	2223 (15.49)	8892 (15.49)		1742 (15.41)	1742 (15.41)	
70–74	3942 (27.47)	15,768 (27.47)		3102 (27.45)	3102 (27.45)	
75+	1308 (9.12)	5232 (9.12)		1043 (9.23)	1043 (9.23)	
Sex (n, %)			0.00			0.00
Male	11,329 (78.96)	45,316 (78.96)		8905 (78.81)	8905 (78.81)	
Female	3019 (21.04)	12,076 (21.04)		2395 (21.19)	2395 (21.19)	
Income (n, %)			0.00			0.00
1 (lowest)	3307 (23.05)	13,228 (23.05)		2596 (22.98)	2596 (22.98)	
2	2696 (18.79)	10,784 (18.79)		2120 (18.76)	2120 (18.76)	
3	2094 (14.59)	8376 (14.59)		1653 (14.63)	1653 (14.63)	
4	2693 (18.77)	10,772 (18.77)		2122 (18.78)	2122 (18.78)	
5 (highest)	3558 (24.80)	14,232 (24.80)		2809 (24.86)	2809 (24.86)	
Region of residence (n, %)			0.00			0.00
Urban	6095 (42.48)	24,380 (42.48)		4796 (42.45)	4796 (42.45)	
Rural	8253 (57.52)	33,012 (57.52)		6503 (57.55)	6503 (57.55)	
Obesity † (n, %)			0.16			0.00
Underweight	719 (5.01)	1579 (2.75)		486 (4.30)	486 (4.30)	
Normal	5382 (37.51)	19,076 (33.24)		4146 (36.69)	4146 (36.69)	
Overweight	3625 (25.26)	15,531 (27.06)		2910 (25.76)	2910 (25.76)	
Obese I	4184 (29.16)	19,210 (33.47)		3402 (30.11)	3402 (30.11)	
Obese II	438 (3.05)	1996 (3.48)		356 (3.15)	356 (3.15)	
Smoking status (n, %)			0.14			0.00
Non-smoker	5975 (41.64)	28,156 (49.06)		4879 (43.18)	4879 (43.18)	
Past smoker	4571 (31.86)	16,641 (29.00)		3545 (31.37)	3545 (31.37)	
Current smoker	3802 (26.50)	12,595 (21.95)		2876 (25.45)	2876 (25.45)	
Alcohol consumption (n, %)			0.00			0.00
<1 time a week	7795 (54.33)	31,221 (54.40)		6152 (54.44)	6152 (54.44)	
≥1 time a week	6553 (45.67)	26,171 (45.60)		5148 (45.56)	5148 (45.56)	
SBP (mean, SD)	126.74 (15.48)	127.22 (15.22)	0.03	126.83 (13.69)	126.83 (6.80)	0.00
DBP (mean, SD)	77.15 (10.18)	77.29 (9.91)	0.01	77.18 (9.01)	77.18 (4.42)	0.00
FBG (mean, SD)	105.81 (29.96)	105.41 (28.23)	0.01	105.68 (26.29)	105.68 (12.95)	0.00
Total cholesterol (mean, SD)	189.29 (43.37)	190.35 (40.48)	0.03	189.60 (38.70)	189.60 (17.93)	0.00
CCI score (mean, SD)	0.80 (1.15)	0.58 (1.09)	0.20	0.75 (0.97)	0.75 (0.57)	0.00
Gout (n, %)			0.02			0.02
No	13,615 (94.89)	54,718 (95.34)		10,723 (94.90)	10,780 (95.40)	
Yes	733 (5.11)	2674 (4.66)		577 (5.10)	520 (4.60)	

Abbreviations: CCI, Charlson Comorbidity Index; SBP, systolic blood pressure; DBP, diastolic blood pressure; FBG, fasting blood glucose; † obesity (BMI, body mass index, kg/m^2^) was categorized as < 18.5 (underweight), ≥ 18.5 to < 23 (normal), ≥ 23 to < 25 (overweight), ≥ 25 to < 30 (obese I), and ≥ 30 (obese II).

**Table 2:** Crude and overlap-propensity-score-weighted odds ratios of gout for head and neck cancer.

Characteristics	N of Case	N of Control	Odds Ratios (95% Confidence Interval)			
	(Exposure/Total, %)	(Exposure/Total, %)	Crude	*p*-Value	Overlap-Weighted Model †	*p*-Value
Odds ratios for head and neck cancer (Total)						
Non-gout	13,615/14,348 (94.9)	54,718/57,392 (95.3)	1		1	
Gout	733/14,348 (5.1)	2674/57,392 (4.7)	1.10 (1.01–1.20)	0.024 *	1.12 (1.04–1.20)	0.002 *
Odds ratios for oral cavity cancer						
Non-gout	3853/4061 (94.9)	64,480/67,679 (95.3)	1		1	
Gout	208/4061 (5.1)	3199/67,679 (4.7)	1.09 (0.94–1.26)	0.250	1.25 (1.16–1.34)	<0.001 *
Odds ratios for oropharynx cancer						
Non-gout	2175/2295 (94.8)	66,158/69,445 (95.3)	1		1	
Gout	120/2295 (5.2)	3287/69,445 (4.7)	1.11 (0.92–1.34)	0.272	1.08 (1.01–1.15)	0.029 *
Odds ratios for nasopharynx cancer						
Non-gout	1427/1484 (96.2)	66,906/70,256 (95.2)	1		1	
Gout	57/1484 (3.8)	3350/70,256 (4.8)	0.80 (0.61–1.04)	0.097	0.89 (0.83–0.96)	0.003 *
Odds ratios for hypopharynx cancer						
Non-Gout	1409/1494 (94.3)	66,924/70,246 (95.3)	1		1	
Gout	85/1494 (5.7)	3322/70,246 (4.7)	1.22 (0.97–1.52)	0.084	1.04 (0.98–1.11)	0.200
Odds ratios for nasal cavity/sinus cancer						
Non-gout	1228/1272 (96.5)	67,105/70,468 (95.2)	1		1	
Gout	44/1272 (3.5)	3363/70,468 (4.8)	0.72 (0.53–0.97)	0.030 *	0.78 (0.72–0.84)	<0.001 *
Odds ratios for larynx cancer						
Non-gout	3885/4143 (93.8)	64,448/67,597 (95.3)	1		1	
Gout	258/4143 (6.2)	3149/67,597 (4.7)	1.36 (1.19–1.55)	<0.001 *	1.12 (1.06–1.20)	<0.001 *
Odds ratios for salivary gland cancer						
Non-gout	1675/1732 (96.7)	66,658/70,008 (95.2)	1		1	
Gout	57/1732 (3.3)	3350/70,008 (4.8)	0.68 (0.52–0.88)	0.004 *	0.88 (0.81–0.96)	0.002 *

* significance at *p* < 0.05. † Adjusted for age, sex, income, region of residence, SBP, DBP, FBG, total cholesterol, obesity, smoking, alcohol consumption, and CCI scores.

## Data Availability

The datasets presented in this article are not readily available because the data were derived from Korean Health Insurance system. Requests to access the datasets should be directed to NHIS-NSC.

## Supplementary Materials

The following supporting information can be downloaded at: https://www.mdpi.com/article/10.3390/jcm13113136/s1, Table S1. Subgroup analysis of crude and overlap propensity score weighted odds ratios of gout for head and neck cancer according to age and sex. Table S2. Subgroup analysis of crude and overlap propensity score weighted odds ratios of gout for oral cavity cancer according to age and sex. Table S3. Subgroup analysis of crude and overlap propensity score weighted odds ratios of gout for oropharynx cancer according to age and sex. Table S4. Subgroup analysis of crude and overlap propensity score weighted odds ratios of gout for nasopharynx cancer according to age and sex. Table S5. Subgroup analysis of crude and overlap propensity score weighted odds ratios of gout for hypopharynx cancer according to age and sex. Table S6. Subgroup analysis of crude and overlap propensity score weighted odds ratios of gout for nasal cavity/sinus cancer according to age and sex. Table S7. Subgroup analysis of crude and overlap propensity score weighted odds ratios of gout for larynx cancer according to age and sex.
