# Supplementary material for: Association of Gout with Head and Neck Cancer: Longitudinal Follow-Up Studies Using a National Health Insurance Database in South Korea
Source: J Clin Med. 2024 May 27;13(11):3136. doi: 10.3390/jcm13113136 (PMC11173149; doi:10.3390/jcm13113136)
Supplement: Supplementary file 1 [file jcm-13-03136-s001.zip › jcm-2987140-supplementary.pdf]

Table S1. Subgroup analyse of crude and overlap propensity score weighted odd ratios of Gout for head and neck cancer according to age and sex.

Characteristics	N of HNC	N of Control	Odd ratios (95% confidence interval)			
	(exposure/total, %)	(exposure/total, %)	Crude	P-value	Overlap weighted model †	P-value
Age < 65 years old						
Non-Gout	6,604/6,875 (96.1)	26,521/27,500 (96.4)	1		1	
Gout	271/6,875 (3.9)	979/27,500 (3.6)	1.11 (0.97-1.28)	0.131	1.11 (0.90-1.36)	0.320
Age ≥ 65 years old						
Non-Gout	7,011/7,473 (93.8)	28,197/29,892 (94.3)	1		1	
Gout	462/7,473 (6.2)	1,695/29,892 (5.7)	1.10 (0.99-1.22)	0.090	1.13 (0.97-1.33)	0.111
Male						
Non-Gout	10,644/11,329 (94.0)	42,788/45,316 (94.4)	1		1	
Gout	685/11,329 (6.0)	2,528/45,316 (5.6)	1.09 (1.00-1.19)	0.054	1.11 (0.98-1.26)	0.101
Female						
Non-Gout	2,971/3,019 (98.4)	11,930/12,076 (98.8)	1		1	
Gout	48/3,019 (1.6)	146/12,076 (1.2)	1.32 (0.95-1.83)	0.097	1.27 (0.78-2.07)	0.337

Abbreviations: SBP, Systolic blood pressure; DBP, Diastolic blood pressure; FBG. Fasting blood glucose; CCI, Charlson Comorbidity Index;

* Significance at P < 0.05

† Adjusted for age, sex, income, region of residence, SBP, DBP, FBG, total cholesterol, obesity, smoking, alcohol consumption, CCI scores

Table S2. Subgroup analyse of crude and overlap propensity score weighted odd ratios of Gout for oral cavity cancer according to age and sex.

Characteristics	N of oral cavity cancer (exposure/total, %)	N of Control (exposure/total, %)	Odd ratios (95% confidence interval)			
			Crude	P-value	Overlap weighted model †	P-value
Age < 65 years old						
Non-Gout	1,947/2,036 (95.6)	31,178/32,339 (96.4)	1		1	
Gout	89/2,036 (4.4)	1,161/32,339 (3.6)	1.23 (0.98-1.53)	0.068	1.33 (0.94-1.88)	0.102
Age ≥ 65 years old						
Non-Gout	1,906/2,025 (94.1)	33,302/35,340 (94.2)	1		1	
Gout	119/2,025 (5.9)	2,038/35,340 (5.8)	1.02 (0.84-1.23)	0.836	1.24 (0.94-1.64)	0.132
Meal						
Non-Gout	2,547/2,730 (93.3)	50,885/53,915 (94.4)	1		1	
Gout	183/2,730 (6.7)	3,030/53,915 (5.6)	1.21 (1.03-1.41)	0.017*	1.25 (0.99-1.57)	0.056
Female						
Non-Gout	1,306/1,331 (98.1)	13,595/13,764 (98.8)	1		1	
Gout	25/1,331 (1.9)	169/13,764 (1.2)	1.54 (1.01-2.35)	0.046*	1.46 (0.75-2.83)	0.262

Abbreviations: SBP, Systolic blood pressure; DBP, Diastolic blood pressure; FBG. Fasting blood glucose; CCI, Charlson Comorbidity Index;

* Significance at P < 0.05

† Adjusted for age, sex, income, region of residence, SBP, DBP, FBG, total cholesterol, obesity, smoking, alcohol consumption, CCI scores

Table S3. Subgroup analyse of crude and overlap propensity score weighted odd ratios of Gout for oropharynx cancer according to age and sex.

Characteristics	N of oropharynx cancer (exposure/total, %)	N of Control (exposure/total, %)	Odd ratios (95% confidence interval)			
			Crude	P-value	Overlap weighted model †	P-value
Age < 65 years old						
Non-Gout	1,175/1,227 (95.8)	31,950/33,148 (96.4)	1		1	
Gout	52/1,227 (4.2)	1,198/33,148 (3.6)	1.18 (0.89-1.57)	0.252	1.07 (0.70-1.62)	0.760
Age ≥ 65 years old						
Non-Gout	1,000/1,068 (93.6)	34,208/36,297 (94.2)	1		1	
Gout	68/1,068 (6.4)	2,089/36,297 (5.8)	1.11 (0.87-1.43)	0.398	1.09 (0.76-1.57)	0.639
Meal						
Non-Gout	1,861/1,978 (94.1)	51,571/54,667 (94.3)	1		1	
Gout	117/1,978 (5.9)	3,096/54,667 (5.7)	1.05 (0.87-1.27)	0.635	1.10 (0.83-1.45)	0.507
Female						
Non-Gout	314/317 (99.1)	14,587/14,778 (98.7)	1		1	
Gout	3/317 (0.9)	191/14,778 (1.3)	0.73 (0.23-2.30)	0.591	0.66 (0.14-3.11)	0.601

Abbreviations: SBP, Systolic blood pressure; DBP, Diastolic blood pressure; FBG. Fasting blood glucose; CCI, Charlson Comorbidity Index;

* Significance at P < 0.05

† Adjusted for age, sex, income, region of residence, SBP, DBP, FBG, total cholesterol, obesity, smoking, alcohol consumption, CCI scores

Table S4. Subgroup analyse of crude and overlap propensity score weighted odd ratios of Gout for nasopharynx cancer according to age and sex.

Characteristics	N of nasopharynx cancer (exposure/total, %)	N of Control (exposure/total, %)	Odd ratios (95% confidence interval)			
			Crude	P-value	Overlap weighted model †	P-value
Age < 65 years old						
Non-Gout	909/936 (97.1)	32,216/33,439 (96.3)	1		1	
Gout	27/936 (2.9)	1,223/33,439 (3.7)	0.78 (0.53-1.15)	0.214	0.79 (0.46-1.35)	0.388
Age ≥ 65 years old						
Non-Gout	518/548 (94.5)	34,690/36,817 (94.2)	1		1	
Gout	30/548 (5.5)	2,127/36,817 (5.8)	0.94 (0.65-1.37)	0.763	1.03 (0.61-1.74)	0.915
Meal						
Non-Gout	1,120/1,173 (95.5)	52,312/55,472 (94.3)	1		1	
Gout	53/1,173 (4.5)	3,160/55,472 (5.7)	0.78 (0.59-1.03)	0.085	0.90 (0.61-1.32)	0.588
Female						
Non-Gout	307/311 (98.7)	14,594/14,784 (98.7)	1		1	
Gout	4/311 (1.3)	190/14,784 (1.3)	1.00 (0.37-2.71)	1.000	1.10 (0.25-4.95)	0.897

Abbreviations: SBP, Systolic blood pressure; DBP, Diastolic blood pressure; FBG. Fasting blood glucose; CCI, Charlson Comorbidity Index;

* Significance at $P < 0.05$

† Adjusted for age, sex, income, region of residence, SBP, DBP, FBG, total cholesterol, obesity, smoking, alcohol consumption, CCI scores

Table S5. Subgroup analyse of crude and overlap propensity score weighted odd ratios of Gout for hypopharynx cancer according to age and sex.

Characteristics	N of hypopharynx cancer	N of Control	Odd ratios (95% confidence interval)			
	(exposure/total, %)	(exposure/total, %)	Crude	P-value	Overlap weighted model †	P-value
Age < 65 years old						
Non-Gout	439/451 (97.3)	32,686/33,924 (96.4)	1		1	
Gout	12/451 (2.7)	1,238/33,924 (3.6)	0.72 (0.41-1.29)	0.268	0.69 (0.32-1.50)	0.352
Age ≥ 65 years old						
Non-Gout	970/1,043 (93.0)	34,238/36,322 (94.3)	1		1	
Gout	73/1,043 (7.0)	2,084/36,322 (5.7)	1.24 (0.97-1.58)	0.085	1.15 (0.80-1.65)	0.443
Meal						
Non-Gout	1,321/1,405 (94.0)	52,111/55,240 (94.3)	1		1	
Gout	84/1,405 (6.0)	3,129/55,240 (5.7)	1.06 (0.85-1.32)	0.615	1.04 (0.75-1.44)	0.802
Female						
Non-Gout	88/89 (98.9)	14,813/15,006 (98.7)	1		1	
Gout	1/89 (1.1)	193/15,006 (1.3)	0.87 (0.12-6.29)	0.892	0.79 (0.05-13.1)	0.866

Abbreviations: SBP, Systolic blood pressure; DBP, Diastolic blood pressure; FBG. Fasting blood glucose; CCI, Charlson Comorbidity Index;

* Significance at P < 0.05

† Adjusted for age, sex, income, region of residence, SBP, DBP, FBG, total cholesterol, obesity, smoking, alcohol consumption, CCI scores

Table S6. Subgroup analyse of crude and overlap propensity score weighted odd ratios of Gout for nasal cavity/sinus cancer according to age and sex.

Characteristics	N of nasal cavity/sinus cancer (exposure/total, %)	N of Control (exposure/total, %)	Odd ratios (95% confidence interval) Crude	P-value	Overlap weighted model †	P-value
Age < 65 years old						
Non-Gout	630/644 (97.8)	32,495/33,731 (96.3)	1		1	
Gout	14/644 (2.2)	1,236/33,731 (3.7)	0.58 (0.34-1.00)	0.048*	0.61 (0.30-1.24)	0.172
Age ≥ 65 years old						
Non-Gout	598/628 (95.2)	34,610/36,737 (94.2)	1		1	
Gout	30/628 (4.8)	2,127/36,737 (5.8)	0.82 (0.56-1.18)	0.282	0.91 (0.54-1.52)	0.718
Meal						
Non-Gout	848/887 (95.6)	52,584/55,758 (94.3)	1		1	
Gout	39/887 (4.4)	3,174/55,758 (5.7)	0.76 (0.55-1.05)	0.099	0.77 (0.50-1.19)	0.245
Female						
Non-Gout	380/385 (98.7)	14,521/14,710 (98.7)	1		1	
Gout	5/385 (1.3)	189/14,710 (1.3)	1.01 (0.41-2.47)	0.981	0.91 (0.25-3.28)	0.890

Abbreviations: SBP, Systolic blood pressure; DBP, Diastolic blood pressure; FBG. Fasting blood glucose; CCI, Charlson Comorbidity Index;

* Significance at P < 0.05

† Adjusted for age, sex, income, region of residence, SBP, DBP, FBG, total cholesterol, obesity, smoking, alcohol consumption, CCI scores

Table S7. Subgroup analyse of crude and overlap propensity score weighted odd ratios of Gout for larynx cancer according to age and sex.

Characteristics	N of larynx cancer	N of Control	Odd ratios (95% confidence interval)			
	(exposure/total, %)	(exposure/total, %)	Crude	P-value	Overlap weighted model †	P-value
Age < 65 years old						
Non-Gout	1,449/1,533 (94.5)	31,676/32,842 (96.4)	1		1	
Gout	84/1,533 (5.5)	1,166/32,842 (3.6)	1.57 (1.25-1.98)	<0.001*	1.30 (0.92-1.84)	0.140
Age ≥ 65 years old						
Non-Gout	2,436/2,610 (93.3)	32,772/34,755 (94.3)	1		1	
Gout	174/2,610 (6.7)	1,983/34,755 (5.7)	1.18 (1.01-1.39)	0.042*	1.06 (0.84-1.34)	0.623
Meal						
Non-Gout	3,667/3,922 (93.5)	49,765/52,723 (94.4)	1		1	
Gout	255/3,922 (6.5)	2,958/52,723 (5.6)	1.17 (1.03-1.34)	0.020*	1.12 (0.92-1.36)	0.242
Female						
Non-Gout	218/221 (98.6)	14,683/14,874 (98.7)	1		1	
Gout	3/221 (1.4)	191/14,874 (1.3)	1.06 (0.34-3.34)	0.921	1.08 (0.21-5.53)	0.927

Abbreviations: SBP, Systolic blood pressure; DBP, Diastolic blood pressure; FBG. Fasting blood glucose; CCI, Charlson Comorbidity Index;

* Significance at P < 0.05

† Adjusted for age, sex, income, region of residence, SBP, DBP, FBG, total cholesterol, obesity, smoking, alcohol consumption, CCI scores
